# Swimming mechanics and propulsive efficiency in the chambered nautilus

**DOI:** 10.1098/rsos.170467

**Published:** 2018-02-21

**Authors:** Thomas R. Neil, Graham N. Askew

**Affiliations:** School of Biomedical Sciences, Faculty of Biological Sciences, University of Leeds, Leeds LS2 9JT, UK

**Keywords:** fluid dynamics, wake structure, vorticity, cephalopod, mollusc

## Abstract

The chambered nautilus (*Nautilus pompilius*) encounters severe environmental hypoxia during diurnal vertical movements in the ocean. The metabolic cost of locomotion (*C*_met_) and swimming performance depend on how efficiently momentum is imparted to the water and how long on-board oxygen stores last. While propulsive efficiency is generally thought to be relatively low in jet propelled animals, the low *C*_met_ in *Nautilus* indicates that this is not the case. We measured the wake structure in *Nautilus* during jet propulsion swimming, to determine their propulsive efficiency. Animals swam with either an anterior-first or posterior-first orientation. With increasing swimming speed, whole cycle propulsive efficiency increased during posterior-first swimming but decreased during anterior-first swimming, reaching a maximum of 0.76. The highest propulsive efficiencies were achieved by using an asymmetrical contractile cycle in which the fluid ejection phase was relatively longer than the refilling phase, reducing the volume flow rate of the ejected fluid. Our results demonstrate that a relatively high whole cycle propulsive efficiency underlies the low *C*_met_ in *Nautilus*, representing a strategy to reduce the metabolic demands in an animal that spends a significant part of its daily life in a hypoxic environment.

## 1. Background

Chambered nautilus (*Nautilus pompilius*) perform diurnal vertical movements involving depth changes of 500–600 m. During the day they either rest at depths of around 200 m or forage at depths up to 700 m, and during the night move almost continuously between depths of 130 and 700 m [1]. While foraging at depth animals encounter low concentrations of oxygen (oxygen partial pressure, *P*O_2_, approximately 50 mmHg or 30% of air-equilibrated surface water; [2].) The high capacity of the haemolymph for oxygen storage [3], the high affinity of haemocyanin for oxygen [4,5], the ability to extract ambient oxygen via the superficial capillaries in the absence of gill perfusion, and the ability to use oxygen stored in the shell chambers [6] are physiological adaptations that enable *Nautilus* to not only survive hypoxia but to also maintain sufficient metabolic scope to perform their extensive vertical migrations [7]. It is only at *P*O_2_ below 50 mmHg, encountered in oxygen deficient water or during retraction of the animal into its shell, that metabolic suppression is required to protect against hypoxia [7].

**Figure 1.**
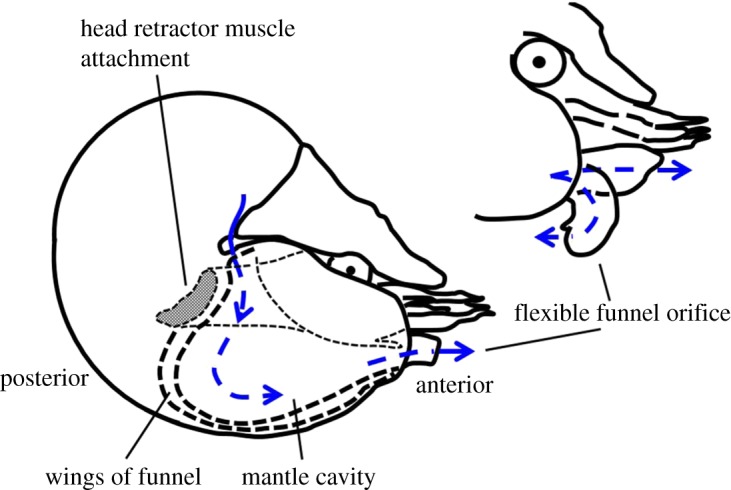
Mechanisms of producing jets during swimming in *Nautilus*: (1) by the contraction of the large head retractor muscle; (2) through the rhythmic contraction of the funnel wings. Inset demonstrates how the flexible jet orifice—the funnel—can move to direct water in multiple directions. Blue lines represent the flow of fluid through the animal which facilitates both oxygen exchange and locomotion. Figure adapted from [9,10].

A further adaptation that supports hypoxia tolerance relates to their economical locomotion [8]. *Nautilus* swims by jet propulsion. Powerful jetting is produced by the compression of the mantle cavity produced by synchronous contraction of the retractor and funnel muscles (figure 1; [9,10]). Compression of the mantle cavity results in a pressure difference between the mantle cavity and the ambient water, expelling water from the mantle cavity via the funnel or siphon (as well as along the top edges of the shell aperture during very powerful contractions) [9,11]. Slower swimming movements and ventilation are powered by rhythmic contractions of the funnel flaps that result in a wave of movement that moves anteriorly along the funnel wings, producing unidirectional flow of water across the gills, through the mantle cavity and exiting through the funnel [11,12]. The fluid jet is formed by the funnel wings that extend along either side of the head and overlap along the ventral side of the animal terminating in the funnel. The manoeuvrable funnel allows the water to be ejected at a range of angles giving *Nautilus* the ability to swim in all directions.

The efficiency of jet propulsion swimming is generally considered to be lower than undulatory swimming [13]. This difference in efficiency originates from the fact that, for a given thrust, jet propulsion swimming involves accelerating a small mass of fluid to a high velocity to achieve propulsion, whereas greater efficiencies are achievable by accelerating a large mass of water at slower velocities, as can occur in an undulatory swimmer [14]. However, despite the presence of an external shell and the use of jet propulsion to power locomotion, *Nautilus* has a lower minimum metabolic cost of locomotion (*C*_met_) compared with squid and, at low speeds, salmon [8]. The low *C*_met_ is advantageous in conserving the limited oxygen stores when swimming at depth in hypoxic conditions. The efficiency with which muscular work is transferred to useful hydrodynamic work is one step in the transduction of chemical energy into useful work in the environment. Therefore, knowledge of the wake structure may give some insights into the low cost of jet propulsion swimming in *Nautilus*. For example, *Nautilus* may be able to manipulate the structure of the jet, enhancing the propulsive efficiency as has been observed in jets produced using mechanical pistons [15]. The aim of this study was to measure the wake structure of *Nautilus* during jet propulsion swimming, and to determine their propulsive efficiency. It was hypothesized that *Nautilus* would have a high whole cycle propulsive efficiency compared to other jet propelled organisms consistent with the requirement for an economical lifestyle of an animal living in a hypoxic environment.

## 2. Material and methods

### 2.1. Animals

Chambered nautilus (*Nautilus pompilius* Linnaeus, 1758, *n* = 5) were obtained from a marine livestock supplier (Tropical Marine Centre, Manchester, UK) and housed in a 250 l aquarium in artificial seawater (Instant Ocean, Aquarium Systems, Inc.). The aquarium was maintained at a temperature of 17°C and a salinity of 34 ppt. *Nautilus* were fed twice weekly with whole shrimp.

### 2.2. Wake visualization and analysis

Visualization of the wake structure took place in a 126 l (610 × 460 × 450 mm, length × width × height) glass aquarium containing artificial seawater at a temperature and salinity matching that of the holding tank. *Nautilus* were transferred to the experimental tank and allowed at least 15 min to adjust to their surroundings. As *Nautilus* are olfactory foragers [16], a shrimp was added to the water to stimulate swimming, eliciting a variety of swimming behaviours, e.g. anterior or posterior swimming.

Quantitative analysis of the jet structure of *Nautilus* was obtained using two-dimensional particle imaging velocimetry (PIV). The experimental tank was seeded with aluminium oxide (H7881 5 µm, Sigma-Aldrich, Germany; following [17]) at a density of 30 mg l^−1^. Particles were illuminated with a 1 W continuous 532 nm, green laser (Shanghai Dream Lasers Technology Co., Ltd, Shanghai, People's Republic of China) directed through a Powell lens (Thorlabs, Inc., Newton, NJ, USA) creating a 1 mm thick, vertically orientated light sheet. The aim was to visualize the wake of swimming *Nautilus* in the sagittal plane; only those sequences in which the laser bisected the jet orifice and thereby the middle of the vortex structures were used for analysis. The *Nautilus* and particle movements were recorded using a high-speed camera (FASTCAM SA3, Photron USA, San Diego, CA, USA) recording at 500 frame s^−1^, shuttered at 1/500 s^−1^ and recording at a 1024 × 1024 pixel resolution.

The positional data of the illuminated particles were analysed using an open source software (PIVlab v. 1.41 [18]). The image sequences were pre-processed with a contrast-limited adaptive histogram equalization tool to enhance contrast. The body of the *Nautilus* was masked on the images to eliminate edge effects. A cross correlation technique was used with adaptive multi-pass processing to analyse image pairs and to track particle movement between frames. A total of three passes were used to analyse images, with an initial interrogation window of 128 × 128 pixels and a final size of 32 × 32 pixels with a 50% overlap between each pass. A standard deviation filter was used to remove vectors that were more than 7 deviations away from the mean jet flow. An average of 0.43 ± 0.02% of the vectors was found to be erroneous across all swimming sequences. Missing velocity vectors were interpolated using a boundary value solver, giving a smooth interpolation that tended towards the average boundary velocities. The range of pixel displacements across interrogation windows was 2–7 pixels with higher pixel displacement occurring at the start of the jetting sequence.

Jet thrust, *T*, is the force propelling the animal and equals the rate of change of momentum in the surrounding fluid. Thrust was calculated as (following [19])

2.1 $$T=\rho {\bar{u}}_{j}^{2}A_{j},$$

where *ρ* is seawater density (1025 kg m^−3^), ${\bar{u}}_{j}$ is the average jet velocity (the time average of the average jet core velocity during the jet period) and *A*_j_ is the cross-sectional area of the jet orifice, measured from still images of the jet orifice (ImageJ v. 1.50i, Bethesda, Maryland, USA).

Whole cycle propulsive efficiency (*η*_wc_) is the ratio of useful power to total power (i.e. the sum of useful and wasted power), and was calculated using a method developed for jet propulsion swimming, which accounts for the acceleration of the water during both the refilling (intake) and contraction (expulsion) phases of the swimming cycle [20]. The useful power is the product of the force propelling the animal (the rate of change of momentum, $m_{j}{\bar{u}}_{j}$) and the velocity of the animal ($\bar{U}$), i.e. $m_{j}\bar{U}{\bar{u}}_{j}$. The rate of loss of energy in the wake is $(1/2)m_{j}{\bar{u}}_{j}^{2}$ and the kinetic energy is given to the water entering the mantle cavity at a rate $(1/2)m_{j}{\bar{u}}_{r}^{2}$, giving a total power of $m_{j}\bar{U}{\bar{u}}_{j}+(1/2)m_{j}{\bar{u}}_{r}^{2}+(1/2)m_{j}{\bar{u}}_{j}^{2}$. Therefore, whole cycle efficiency is given by

2.2 $$\eta_{\mathrm{WC}}=\frac{2\bar{U}{\bar{u}}_{j}}{2\bar{U}{\bar{u}}_{j}+{\bar{u}}_{r}^{2}+{\bar{u}}_{j}^{2}},$$

where *Ū* is the time averaged velocity of the animal, $m_{j}$ is the mass of water passing through the animal in unit time, ${\bar{u}}_{r}$ is the refill velocity, i.e. the velocity of the fluid at the intake orifice during the refilling of the mantle. Difficulty in visualizing the flow near the refill orifices of the *Nautilus* meant that refill velocities had to be estimated. It was assumed that the total volume of water ejected during jetting was equal to the volume of water taken in to the mantle during refilling. Therefore, the refill velocity was estimated as

2.3 $${\bar{u}}_{r}=\frac{{\bar{u}}_{j}A_{j}t_{j}}{A_{r}t_{r}},$$

where *A*_r_ is the area of the refill orifice and *t*_j_ and *t*_r_ are the durations of the jetting and refill periods, respectively. Jet duration (*t*_j_) was taken to be the time period between the beginning of contraction of the head into the mantle cavity and the beginning of relaxation of the head to its initial position. The refill duration (*t*_r_) was defined as the period between the onset of relaxation of the head and the start of the next contraction cycle. The sum of these two periods is the total cycle duration (*t*_cd_). Duty cycle was defined as the ratio of *t*_j_ to *t*_cd_. Slip, an indicator of the inverse of propulsive efficiency, was calculated as ${\bar{u}}_{j}/\bar{U}$ [19].

The ratio of jet length to jet diameter was calculated as *L*_j_/*D*_j_, where *L*_j_ is the jet length measured as the extent of the vorticity field along the jet centreline that exceeded the background flow vorticity by 20%, and *D*_j_ is the diameter of the vortex ring measured from the two peaks of vorticity that make up the vortex ring (figure 3*a*; [21]). Approximately, 8–12 vectors were measured across the jet diameter. Mean vorticity was calculated as the mean vorticity of the measured jet length during the contraction phase of the swim cycle.

### 2.3. Statistical analyses

Statistical analysis was carried out in SPSS for Mac (IBM SPSS Statistics for Mac v. 21.0, Armonk, NY, USA). Data were checked for normality using a Shapiro–Wilks test. Linear regressions were fit to the data to test for speed-related changes in swimming mechanics and wake structure. One-way ANOVA was used to test for differences between swimming orientation. Where differences were detected, Tukey's post hoc tests were used to identify where these differences occurred. All data are reported as mean ± s.e.m.

## 3. Results

### 3.1. Swimming behaviour

Morphological data and basic swimming kinematics are reported in electronic supplementary material, table S1. Two distinct swimming orientations were observed, either ‘anterior-first' or ‘posterior-first'. Posterior-first swimming was the most frequently observed with 77% of swims recorded being in this orientation. Average speed during posterior-first swimming was 0.90 ± 0.12 BL s^−1^ (range = 0.35–1.60 BL s^−1^) and 0.73 ± 0.05 BL s^−1^ (range = 0.48–1.19 BL s^−1^) during anterior-first swimming. The average Reynolds number across all swims was 6.9 × 10^3^ (average swimming speed 8.31 cm s^−1^ and shell diameter of 9.24 cm).

**Figure 2.**
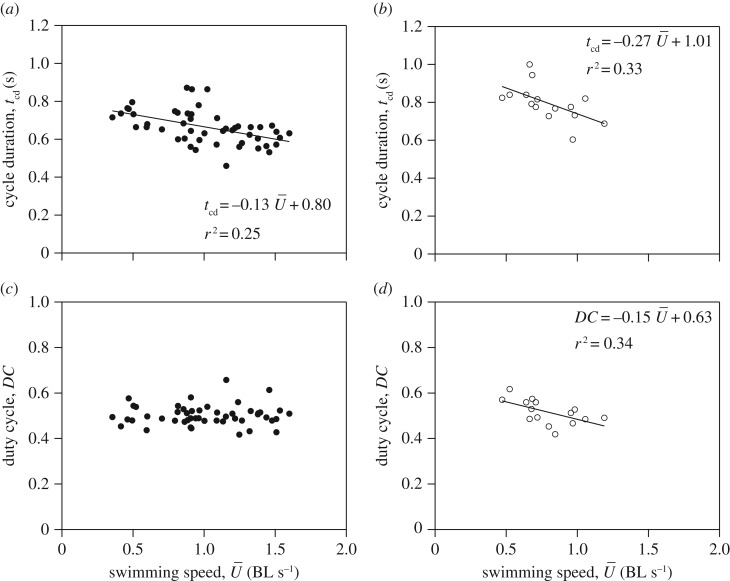
Swimming mechanics as a function of swimming speed in *Nautilus*. Relationship between cycle duration and swimming speed during (*a*) posterior-first and (*b*) anterior-first swimming. The effect of swimming speed on duty cycle during (*c*) posterior-first and (*d*) anterior-first swimming.

Cycle duration decreased with increasing speed during both posterior-first (*F*_1,47_ = 15.28, *p *< 0.001; figure 2*a*) and anterior-first (*F*_1,13_ = 7.01, *p *< 0.05; figure 2*b*) swimming. Duty cycle was 49.73 ± 0.86% (range = 41.72–65.79%) during posterior-first swimming and 51.76 ± 1.12 (range = 41.88–61.72%) during anterior-first swimming. Duty cycle was independent of speed during posterior-first swimming (*p *= 0.91; figure 2*c*) but decreased with increasing speed during anterior-first swimming (*F*_1,13_ = 6.50, *p *< 0.05; figure 2*d*).

### 3.2. Jet wake properties

**Figure 3.**
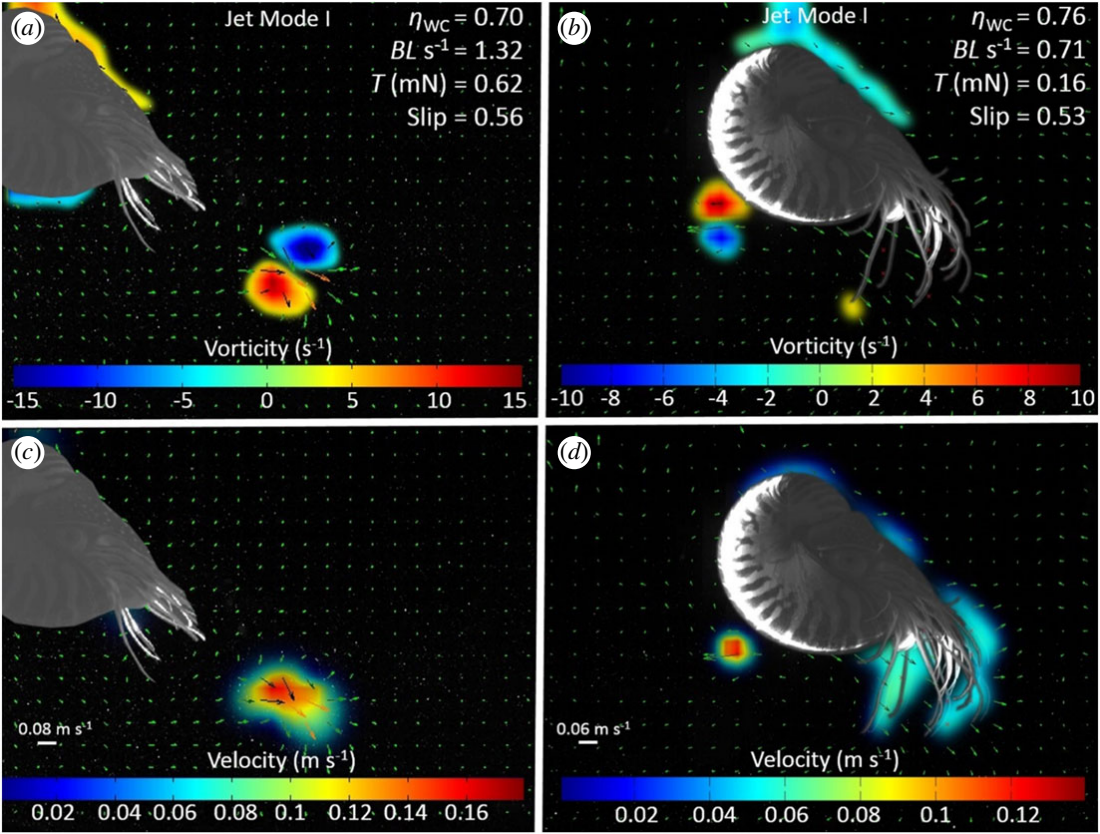
Comparison of instantaneous flow and vorticity between anterior and posterior swimming in *Nautilus* using ‘jet mode 1'. (*a,b*) Vorticity and (*c,d*) velocity vector fields during (*a,c*) posterior and (*b,d*) anterior swimming. Note that the fluid is rolled up into an isolated vortex ring formed, representing ‘jet mode I'. On vorticity plots, red and blue regions denote clockwise and counter-clockwise rotation, respectively. The jet length and jet diameter as represented by the variables *L*_j_ and *D*_j_, are indicated in (*a*) and are defined in the text.

**Figure 4.**
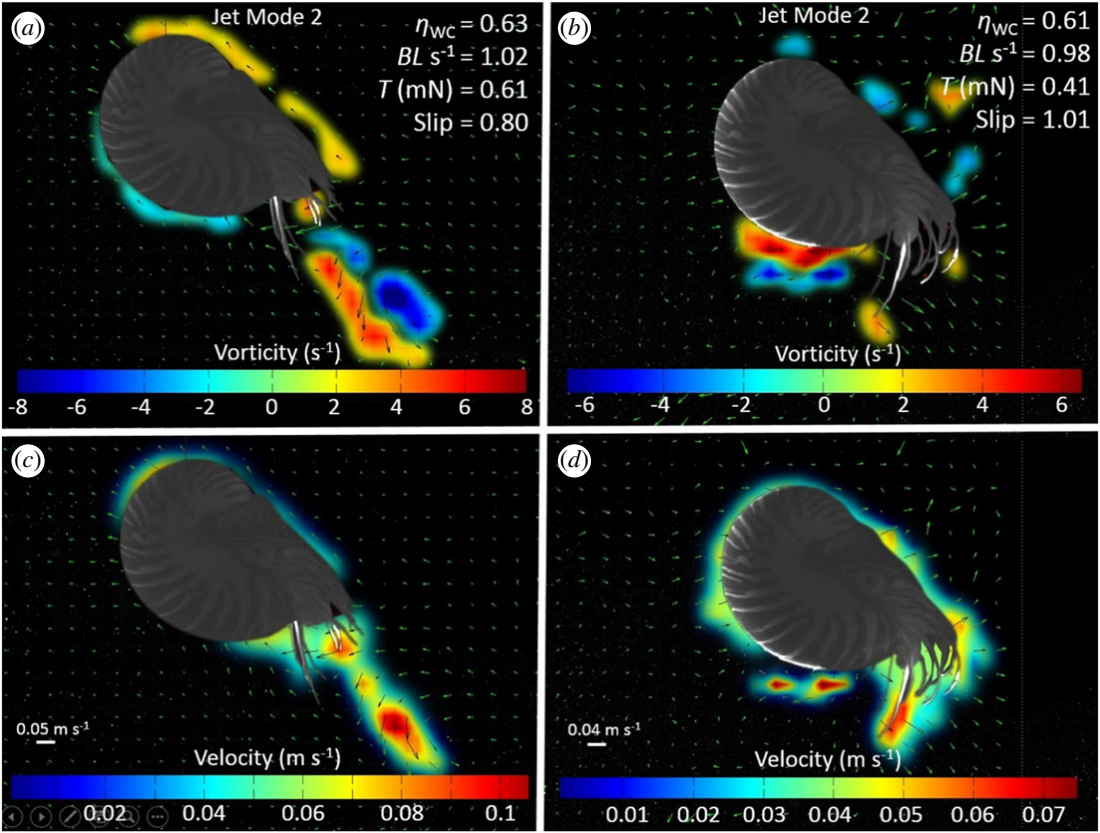
Comparison of instantaneous flow and vorticity between anterior and posterior swimming in *Nautilus* using ‘jet mode 2'. (*a,b*) Vorticity and (*c,d*) velocity vector fields during (*a,c*) posterior and (*b,d*) anterior swimming. Note the presence of a trailing jet that results from vortex pinch-off, representing ‘jet mode 2'. On vorticity plots, red and blue regions denote clockwise and counter-clockwise rotation, respectively.

The two-dimensional divergence was non-zero, indicating the jet structures were not perfectly axisymmetric. The range of variation of two-dimensional divergence was −0.38 to 0.45 s^−1^ for posterior-first swims and −0.17 to 0.26 s^−1^ for anterior-first swims. Two categories of jet structures were observed: jets in which the ejected fluid rolled up into an isolated vortex ring (termed ‘jet mode 1' jets; figure 3; electronic supplementary material, S2); and jets that consisted of an elongated jet of ejected fluid (termed ‘jet mode 2’ jets; figure 4; electronic supplementary material, S2). Both types of jet were observed during both posterior (figure 3*a,c*; figure 4*a*,*c*) and anterior-first (figure 3*b,d*; figure 4*b,d*) swimming behaviours. Both jet modes were observed across the range of speeds during posterior-first swimming. However, during anterior-first swimming, jet mode 1 jets were never seen at speeds exceeding 0.8 BL s^–1^, while jet mode 2 jets were identified at speeds ranging from 0.47 to 1.19 BL s^−1^. The mean area of the jet orifice during refilling was approximately four times the mean jet orifice area during posterior-first swimming and seven times the area during anterior-first swimming (electronic supplementary material, table S1). Refill velocity was estimated to be 0.16–0.36 times the jet velocity.

During posterior-first swimming, *L*_j_/*D*_j_ ranged from 0.79 to 2.16 in jet mode 1 jets and 3.16 to 6.29 in jet mode 2 jets. During anterior-first swimming *L*_j_/*D*_j_ ranged from 1.08 to 1.52 during jet mode 1 swimming and 3.29 to 5.51 during jet mode 2 swimming.

**Figure 5.**
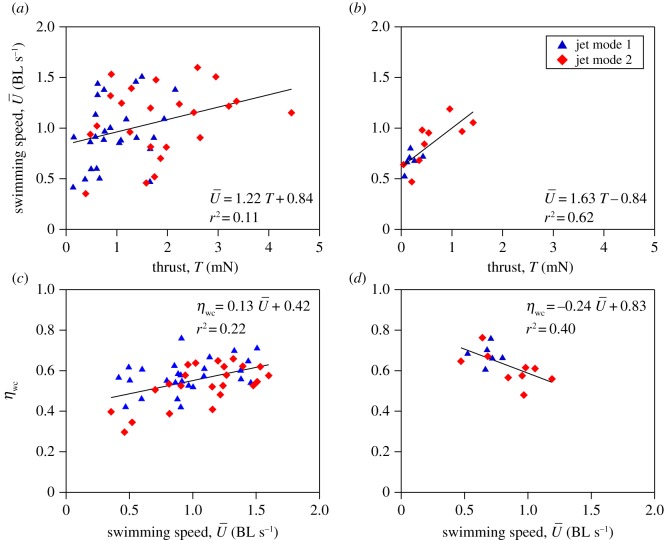
The relationships between jet characteristics and swimming speed in *Nautilus*. Swimming speed plotted as a function of thrust for (*a*) posterior-first and (*b*) anterior-first swimming. Hydrodynamic whole cycle propulsive efficiency *η*_wc_ as a function of swimming speed is illustrated for (*c*) posterior-first and (*d*) anterior-first swimming. The regression lines for all swims combined, are shown.

Mean jet angle relative to swimming trajectory was 16.15 ± 1.58° (range = 1.13–33.69°) in posterior-first swimming and 16.79 ± 2.50° (range = 5.09–32.47°) in anterior-first swimming. Swimming speed was independent of jet angle for both posterior-first (*p *= 0.219) and anterior-first swimming (*p *= 0.138). Swimming speed increased with increasing thrust during both posterior-first (*F*_1,47_ = 5.82, *p *< 0.05; figure 5*a*) and anterior-first swimming (*F*_1,13_ = 23.99, *p *< 0.001 figure 5*b*).

During posterior-first swimming whole cycle propulsive efficiency increased with increasing swimming speed (*F*_1,47_ = 11.46, *p *< 0.05; figure 5*c*). By contrast, whole cycle propulsive efficiency decreased with increasing swimming speed during anterior-first swimming (*F*_1,13_ = 114.53, *p *< 0.05; figure 5*d*). Anterior-first jet mode 1 swimming was more efficient than posterior-first jet mode 2 swimming (*p *< 0.05; electronic supplementary material, figure S1). Thrust varied with swimming orientation and jet mode (*F*_3,9_ = 7.01, *p *< 0.05), with anterior-first jet mode 1 swimming producing less thrust than posterior-first jet mode 2 swimming (*p *< 0.05; electronic supplementary material, figure S1).

## 4. Discussion

### 4.1. Jet modes

Two different categories of wake structure were identified during jet propulsion swimming: jet mode 1 jets, in which all of the ejected fluid rolls up into an isolated vortex ring, and jet mode 2 jets, where fluid is ejected as an elongated jet. These two jet modes are comparable to the categories of jets described, based on two-dimensional recordings similar to those used here, in free-swimming brief squid [21,22] and more recently quantified using three-dimensional particle image velocimetry in the same species [23]. Thrust tended to be higher but whole cycle propulsive efficiency was lower during jet mode 2, compared with jet mode 1, as observed in juvenile and adult squid [21,23]. In *Nautilus* the transition from jet mode 1 to jet mode 2 jets occurred at jet length to diameter ratio (*L*_j_/*D*_j_) of approximately 3 (jet mode 1 *L*_j_/*D*_j_ < 2.16; jet mode 2 *L*_j_/*D*_j_ > 3.16), similar to those observed in other jet propulsion swimmers (e.g. squid, [21]).

### 4.2. Propulsive efficiency

The lack of a difference in whole cycle propulsive efficiency and thrust generation between the two jet modes may explain why the jet mode was not exclusively related to swimming speed in the two swimming orientations. The average whole cycle propulsive efficiency ranged from 0.30 to 0.75 during posterior-first swimming and 0.48 to 0.76 during anterior-first swimming in *Nautilus*. While overlapping, the whole cycle propulsive efficiency in *Nautilus* is higher than the range reported in other jet propelled animals: adult squid (0.42–0.49; [14]), salps (0.47–0.55; [19]), jellyfish (0.09–0.53; [24]). Note that efficiency has not been calculated in the same way in this and previous studies. For example, in jellyfish, the Froude efficiency was calculated [24], which does not include momentum losses during fluid intake and refilling [25]. The calculated jet propulsive efficiency is expected to be higher if the losses during intake and refilling are not included in the calculations [21,26]. In salps, momentum losses during intake and refilling periods are included in the calculation of whole cycle efficiency, though a different equation to that used in this study (assumes refilling occurs passively) was applied [19]. Furthermore, in the study on squid [14], only the horizontal component of the jet was considered to yield ‘useful' work; in our equation 2, the vertical component of the jet is also considered ‘useful', because it contributes to the propulsion of the animal. However, the difference in the calculated whole cycle efficiency is a reduction of only 3.5%, assuming a jet angle of 15°, when considering only the horizontal component of the jet, which alone is insufficient to explain the higher whole cycle propulsive efficiency in *Nautilus*, indicating that the difference is real.

Intriguingly, whole cycle propulsive efficiency increased as a function of speed in posterior-first swimming, but decreased as a function of speed during anterior-first swimming. The relationship between whole cycle propulsive efficiency and swimming orientation may be related to the energy losses associated with re-orienting the funnel during anterior-first swimming. During anterior-first swimming the funnel is turned back on itself, creating a bend through which the fluid must pass before being ejected (figure 1). This may result in energy losses due to turbulence that are proportional to fluid velocity [27], thereby reducing whole cycle propulsive efficiency as swimming speed increases. However, in jet propelling squids, slip tends to decrease with increasing swimming speed, resulting in an increase in efficiency with swimming speed that is independent of swimming orientation [23,26].

An additional or alternative explanation for the inverse relationship between swimming speed and whole cycle propulsive efficiency across swimming orientations could be related to differences in swimming mechanics between the two orientations. In the anterior-first swims, both duty cycle and jet period decreased with increasing swimming speed. Therefore, at their slowest swimming speeds *Nautilus* have asymmetrical contractile cycles, spending more time ejecting fluid than refilling. This results in a relatively low speed jet of fluid being ejected, reducing slip and increasing whole cycle propulsive efficiency [14]. If the area of the orifice through which water is drawn and ejected were the same, the required increase in flow rate during refilling would negate the benefits gained during propulsion. However, during both swimming orientations the size of the refill orifice area is larger (4× during posterior-first swimming and 8× during anterior-first swimming) than during jetting, reducing the refill velocities and avoiding detrimental effects on whole cycle propulsive efficiency. However, as swimming speed increases, the shorter jetting period requires a higher velocity jet, leading to a reduction in whole cycle propulsive efficiency.

In squid, jet angle increases with decreasing swimming speed [26] to provide a vertical force to counteract the negative buoyancy of squid. In squid, this vertical component of the jet reduces the proportion of the jet's momentum that propels the animal forward [28]. The neutrally buoyant *Nautilus* does not need to generate a vertical force, resulting in the absence of a relationship between swimming speed and jet angle (jet angle simply relates to swimming direction). Consequently, the large range of jet angles observed during swimming simply reflects the control of swimming directions. While *Nautilus* has an advantage over squid in not needing to produce a vertical force at slow speeds, as speed increases the relatively high frontal area of *Nautilus* is expected to have a detrimental effect on swimming performance compared to the streamlined bodies and hydrodynamic lift producing fins of squid [29].

The swimming performance of *Nautilus* is ultimately determined by the transfer of mechanical work by the locomotory muscles into useful hydrodynamic work (i.e. work done against drag) in the jet. The efficiency of this process is the whole cycle propulsive efficiency. The locomotory muscles convert chemical energy, ultimately derived from food, into mechanical work; the efficiency of this energy transfer is the net muscle efficiency ($\eta_{\mathrm{mus}}$). Together, the net muscle efficiency and the whole cycle propulsive efficiency determine the overall locomotor efficiency with which chemical energy is converted into useful work in the environment. Therefore, the relatively high whole cycle efficiency in *Nautilus* will increase the overall locomotor efficiency and will reduce *C*_met_: the magnitude of these effects depends on the muscle efficiency, which was not determined here.

As a component of the overall locomotor efficiency and determinant of *C*_met_, it is not surprising that whole cycle propulsive efficiency is related to ecological niche in jet propelled swimmers. In cephalopod molluscs, the slow swimming *Nautilus* has a higher efficiency than the faster swimming squid [14]; in salps it is the slow swimming species that are the most efficient [19]; and cruising predatory jellyfish have a higher efficiency than those that are ambush predators [24]. These observations indicate that the efficiency with which energy is transferred to the environment is under major selective pressure, but increased whole cycle propulsive efficiency appears to come at the expense of swimming speed.

## 5. Conclusion

*Nautilus* are unique amongst cephalopod molluscs in their ability to tolerate hypoxic conditions, which they encounter when undertaking vertical migrations in the water column [6]. These animals employ a number of different physiological strategies to enable them to survive and remain active in such conditions [6,7,30]. This study shows that *Nautilus* are also able to use biomechanical strategies. When swimming at low speeds with an anterior-first orientation, their high whole cycle propulsive efficiency corresponds to a low *C*_met_ [8]. Reducing the metabolic cost of swimming through a high whole cycle propulsive efficiency conserves on-board oxygen supplies and helps avoid anaerobiosis [30].

## Supplementary Material

Nautilus ESM
