# Supplementary material for: Swimming mechanics and propulsive efficiency in the chambered nautilus
Source: R Soc Open Sci. 2018 Feb 21;5(2):170467. doi: 10.1098/rsos.170467 (PMC5830708; doi:10.1098/rsos.170467)
Supplement: Nautilus ESM [file rsos170467supp1.pdf]

Electronic supplementary material

Supplementary Figure S1. Swimming efficiency and thrust during swimming as function of jet mode in *Nautilus.*
Hydrodynamic propulsive efficiency (η_{wc}) as a function of jet mode for posterior-first and anterior-first swimming (A). Thrust as a function of jet mode for posterior-first and anterior-first swimming (B). Error bars represent ±1 s.e.m. Anterior-first jet mode 1 swimming was more efficient than posterior-first jet mode 2 swimming ($p<0.05$). Thrust varied with swimming orientation and jet mode ($F_{3,9}=7.01$, $p<0.05$). Anterior-first jet mode 1 swimming produced less thrust than posterior-first jet mode 2 swimming ($p<0.05$).

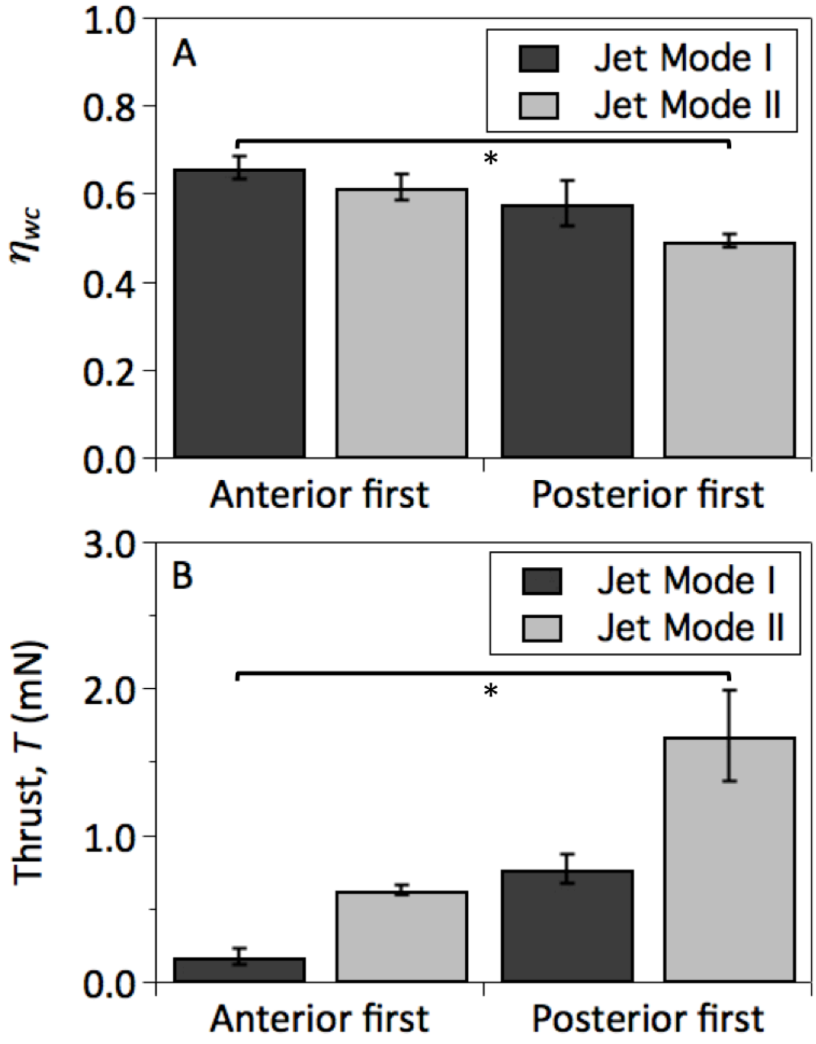

Supplementary Figure S2. Vorticity fields during jet propulsion swimming. Two swimming orientations are shown: posterior (A,B) and anterior swimming (C,D). Red and blue regions denote clockwise and counter-clockwise rotation, respectively. *t* = 0 is the start of fluid ejection in each case.

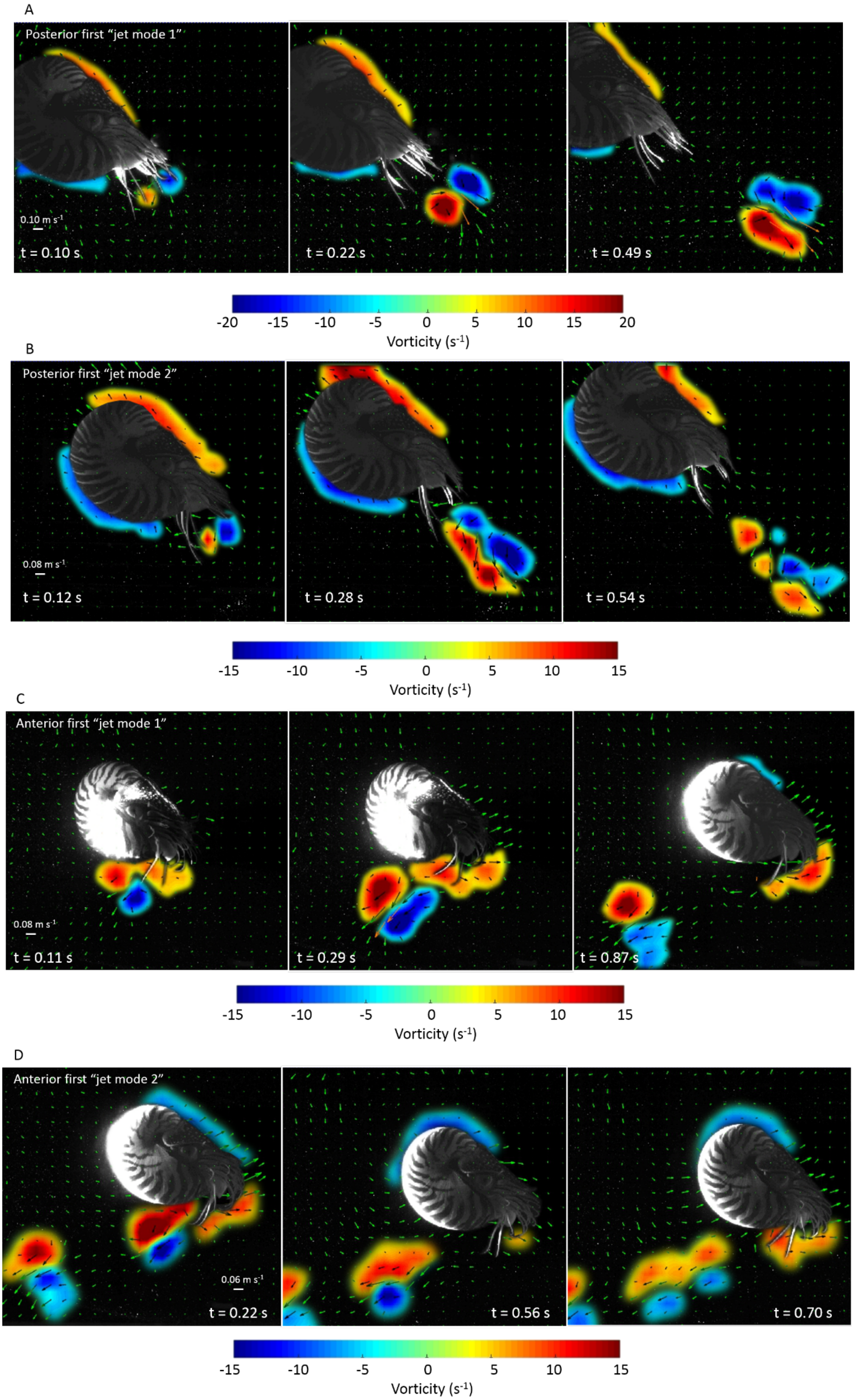

Supplementary Table S1. ***Nautilus*** **morphology and swimming kinematics.**

	Posterior-first	Anterior-first
Body length[a], *BL* (cm)	9.15 ± 0.58	9.28 ± 0.6
Swimming speed[b] ($BL\ s^{-1}$)	0.90 ± 0.12	0.73 ± 0.05
Jet orifice area (cm^2)	1.03 ± 0.08	0.58 ± 0.15
Jet refill orifice area (cm^2)	3.75 ± 0.43	3.98 ± 0.83
Cycle frequency (Hz)	1.51 ± 0.05	1.21 ± 0.06
Duty Cycle[c]	0.51 ± 0.01	0.52 ± 0.01

[a]measured as shell diameter

[b]speed measured relative to body length (*BL*)

[c]proportion of the swimming cycle comprising the power stroke
